# Supplementary material for: Evaluation of Chemical and Physical Triggers for Enhanced Photosynthetic Glycerol Production in Different Dunaliella Isolates
Source: Microorganisms. 2024 Jun 28;12(7):1318. doi: 10.3390/microorganisms12071318 (PMC11278730; doi:10.3390/microorganisms12071318)
Supplement: Supplementary file 1 [file microorganisms-12-01318-s001.zip › microorganisms-3049049-supplementary.pdf]

Supplementary

Table S1: Location of isolation of the algae strains.

Description	Abbreviation	Place of Isolation
Dunaliella tertiolecta	#DT	The Outer Oslofjord, Norway<br>59°28'07.7"N 10°31'37.9"E
Dunaliella sp. 6	#6	Tatiara Water, Santo, South Australia 5264, Australia<br>S 36°09.6874', E 139°38.8328'
Dunaliella sp. 27	#27	Lake Colac, Colac Victoria 3250, Australia<br>S 38°19.9009', E 143°35.2498'
Dunaliella sp. 37	#37	Lake Colac, Colac Victoria 3250, Australia<br>S 38°19.9009', E 143°35.2498'
Dunaliella sp. 83	#83	The Coorong, Coorong South Australia 5264, Australia<br>S 36°30.7013' , E 139°49.5774'
Dunaliella sp. 96	#96	The Coorong, Coorong South Australia 5264, Australia<br>36°30'42.1"S 139°49'34.6"E
Dunaliella sp. 101	#101	Tatiara Water, Santo, South Australia 5264, Australia<br>S 36°09.6874' , E 139°38.8328'
Dunaliella sp. 127	#127	Lake St Clair, Nora Creina South Australia 5276, Australia<br>S 37.296944', E 139.887181'

Table S2: FASTA sequence of the 18S gene sequence of all tested *Dunaliella* strains

> #006_18S
GGAAGCTGCTAAGATTAAGCCATGCATGTCTAAGTATAAACTGCTTATACTGTGAAACTGCGAATGGC
TCATTAAATCAGTTATAGTTTATTTGATGGTACCTTTACTCGGATAACCGTAGTAATTCTAGAGCTAATA
CGTGCGTAAATCCCGACTTCTGGAAGGGACGTATTTATTAGATAAAAGGCCAGCCGGGCTTGCCCGAC
TCTTGGCGAATCATGATAACTTCACGAATCGCACGGCTTCGGCCGGCGATGTTTCATTCAAATTTCTGC
CCTATCAACTTTCGATGGTAGGATAGAGGCCTACCATGGTGGTAACGGGTGACGGAGGATTAGGGTTC
GATTCCGGAGAGGGAGCCTGAGAAACGGCTACCACATCCAAGGAAGGCAGCAGGCGCGCAAATTACC
CAATCCCAACACGGGGAGGTAGTGACAATAAATAACAATACCGGGCATTTTTGTCTGGTAATTGGAAT
GAGTACAATCTAAATCCCTTAACGAGTATCCATTGGAGGGCAAGTCTGGTGCCAGCAGCCGCGGTAAT
TCCAGCTCCAATAGCGTATATTTAAGTTGTTGCAGTTAAAAAGCTCGTAGTTGGATTTCGGGTGGGTTG
TAGCGGTCAGCCTTTGGTTAGTACTGCTACGGCCTACCTTTCTGCCGGGGACGAGCTCCTGGGCTTAAC
TGTCTGGGACTCGGAATCGGCGAGGTTACTTTGAGTAAATTAGAGTGTTCAAAGCAAGCATACGCTCT
GAATACATTAGCATGGAATAACACGATAGGACTCTGGCTTATCTTGTTGGTCTGTAAGACCGGAGTAAT
GATTAAGAGGGACAGTCGGGGGCATTCGTATTTCATTGTCAGAGGTGAAATTCTTGGATTTATGAAAG
ACGAACTTCTGCGAAAGCATTTGCCAAGGATGTTTTCATTAATCAAGAACGAAAGTTGGGGGCTCGAA
GACGATTAGATACCGTCGTAGTCTCAACCATAAACGATGCCGACTAGGGATTGGCAGGTGTTTCGTTA
ATGACCCTGCCAGCACCTTATTGAGAAATCAAAGTTTTTGGGTTCCGGGGGGAGTATGGTCGCAGGCT
GAAACTTAAAGCGAATTGACGAACGGCCCCCCAGGGGTGGAGCCTGCGGCTTAATTTGACTCAACCCG
GAAAACTTACCAGGTCAGACCGGGGAGGATTGACGATTGAAAGCTCTTTCTTGATTTGGGGGGGGGTG
CAGGGCCGTCTTATTGGGGGTTGCCTGGCAGGTGAATCCGGAACAAACGGAACCCCACCCGCAAAAAA
GCCCCTCTTCCCCGGCGGCCCCCGATTTTTTAAGGGCAAGGGCGTTTTCCAAGGGAAGTGGCGGAAAA

CAAGGGGTGTAGGCCCTTATTGAGGGGGCCCCGCGCCCCCAACGATATTTTAAGACAACCCTGTGGAA
ACGGGGCGGGAATTTTA

> #27_18S
TGAGCCTGCATGTCTAAGTATAAACTGCTTATACTGTGAAACTGCGAATGGCTCATTAAATCAGTTATA
GTTTATTTGATGGTACCTTTACTCGGATAACCGTAGTAATTCTAGAGCTAATACGTGCGTAAATCCCGA
CTTCTGGAAGGGACGTATTTATTAGATAAAAGGCCAGCCGGGCTTGCCCGACTCTTGGCGAATCATGAT
AACTTCACGAATCGCACGGCTTCGTGCCGGCGATGTTTCATTCAAATTTCTGCCCTATCAACTTTCGATG
GTAGGATAGAGGCCTACCATGGTGGTAACGGGTGACGGAGGATTAGGGTTCGATTCCGGAGAGGGAG
CCTGAGAAACGGCTACCACATCCAAGGAAGGCAGCAGGCGCGCAAATTACCCAATCCCAACACGGGG
AGGTAGTGACAATAAATAACAATACCGGGCATTTTTGTCTGGTAATTGGAATGAGTACAATCTAAATC
CCTTAACGAGTATCCATTGGAGGGCAAGTCTGGTGCCAGCAGCCGCGGTAATTCCAGCTCCAATAGCG
TATATTTAAGTTGTTGCAGTTAAAAAGCTCGTAGTTGGATTTCGGGTGGGTTGTAGCGGTCAGCCTTTG
GTGAGTACTGCTACGGCCCACCTTTCTGCCGGGGACGTGCTCCTGGGCTTAACTGTCCGGGACACGGAA
TCGGCGAGGTTACTTTGAGTAAATTAGAGTGTTCAAAGCAAGCCTACGCTCTGAATACATTAGCATGG
AATAACACGATAGGACTCTGGCTTATCTTGTTGGTCTGTAAGACCGGAGTAATGATTAAGAGGGACAG
TCGGGGGCATTCGTATTTCATTGTCAGAGGTGAAATTCTTGGGATTTATGAAAGACGAACTTCTGCGAA
AGCATTTGCCAAGGATGTTTTCATTAATTCAAGAACGAAAGTTGGGGGCTCGAAGACGATTAGATACC
GTCGTAGTCTCAACCATAAACGATGCCGACTAGGGATTGGCAGGTGTTTCGTTGATGACCCTGCCAGCA
CCTTATGAGAAAATCAAAGTTTTTGGGTTCCGGGGGGAATATGGTCCCAAGGGTTGAAACTTTAAAGG
GAATTTGACGGAAGGGGCCCCCCCCGGGGGGGGAACCCGGGGGGTTAATTTGTTCCCCAACCCGGGGA
AAAATTTACACGGCCCAAAAAGGGGAGAATTGAAAAATTGAAAATCTTTTTTTTTTTTG

> #37_18S
GCGCGAATCTGTCTCAAGATTAAGCCATGCATGTCTAAGTATAAACTGCTTATACTGTGAAACTGCGAA
TGGCTCATTAAATCAGTTATAGTTTATTTGATGGTACCTTTACTCGGATAACCGTAGTAATTCTAGAGCT
AATACGTGCGTAAATCCCGACTTCTGGAAGGGACGTATTTATTAGATAAAAGGCCAGCCGGGCTTGCC
CGACTCTTGGCGAATCATGATAACTTCACGAATCGCACGGCTTCGTGCCGGCGATGTTTCATTCAAATT
TCTGCCCTATCAACTTTCGATGGTAGGATAGAGGCCTACCATGGTGGTAACGGGTGACGGAGGATTAG
GGTTCGATTCCGGAGAGGGAGCCTGAGAAACGGCTACCACATCCAAGGAAGGCACCTGGCGCGCAAA
TTACCCGATCCCAACACGGGGAGGTAGTGACAATAAATAACAATACCGGGCATTTTTGTCTGGTAATT
GGAATGAGTACAATCTAAATCCCTTAACGAGTATCCGTTGTAGGGCTAGTCTGGTGCCTGCAGCCGCG
GTAGATCCAGCTCCAATAGCGTATATTTAAGTTGTTGCAGTTGAAAAGCTCCGTAGATGGATTTCGGGT
GAGTTGTACGAACAGACTTTGCCGAGTACTGCTAACGTGACTACCTTTCATGCCGAGAACGTGACTCCT
GGGCATAAACTGTCACGGTAACCACCGTAATGCGGACGAAGGTATATCTTTTGAAGTAATAGTTACTC
CGTGCATCTTTA

> #83_18S
CGGCGAATGGCTCATTAAATCAGTTATAGTTTATTTGATGGTACCTTTACTCGGATAACCGTAGTAATT
CTAGAGCTAATACGTGCGTAAATCCCGACTTCTGGAAGGGACGTATTTATTAGATAAAAGGCCAGCCG
GGCTTGCCCGACTCTTGGCGAATCATGATAACTTCACGAATCGCACGGCTTCGTGCCGGCGATGTTTCA
TTCAAATTTCTGCCCTATCAACTTTCGATGGTAGGATAGAGGCCTACCATGGTGGTAACGGGTGACGGA
GGATTAGGGTTCGATTCCGGAGAGGGAGCCTGAGAAACGGCTACCACATCCAAGGAAGGCAGCAGGC
GCGCAAATTACCCAATCCCAACACGGGGAGGTAGTGACAATAAATAACAATACCGGGCATTTTTGTCT
GGTAATTGGAATGAGTACAATCTAAATCCCTTAACGAGTATCCATTGGAGGGCAAGTCTGGTGCCAGC
AGCCGCGGTAATTCCAGCTCCAATAGCGTATATTTAAGTTGTTGCAGTTAAAAAGCTCGTAGTTGGATT
TCGGATGGGTTGTAGCGGTCAGCCTTTGGTGAGTACTGCTACGGCCCATCTTTCTGCCGGGGACGTGCT
CCTGGGCTTAACTGTCCGGGACACGGAATCGGCGAGGTTACTTTGAGTAAATTAGAGTGTTCAAAGCA
AGCCTACGCTCTGAATACATTAGCATGGAATAACACGATAGGACTCTGGCTTATCTTGTTGGTCTGTAA
GACCGGAGTAATGATTAAGAGGGACAGTCGGGGGCATTCGTATTTCATTGTCAGAGGTGAAATTCTTG
GGATTTATGAAAGACGAACTTCTGCGAAAGCATTTGCCAAGGATGTTTTCATTAATCAAGAACGAAAG
TTGGGGGCTCGAAGACGATTAGATACCGTCGTAGTCTCAACCATAAACGATGCCAACTAGGGATTGGC

AGGGGTTTTCGTTGATGACCCTGCCGCACCTTATGAGAAATCAAAGTTTTGGGTTCCGGGGGGGGATA
ATGGGCGCCAAGGCTGAAACTTTAAGGGAATTTGACGAGAGGGCCCACCCGGCGGGGGAGCCGCGGG
TTATTTTGATCCACACGGGAAAATTTCAGGCCGCCCCCGGGGGGGATGTTAAAATTGAAACCTTCTGTT
TGTGGGGGGGGGGGGGGGGGGCCCCTCCTTCTGTGGTGGGGGTGTGTTTGGTGTTTTTGTGTAGAAA
AAGAAAACCCCCCGCCGAGAAAAACCCCCCCCCCCGGGGGCCGGCCTTTTATAGGA

> #96_18S
TCTAAGTATAAACTGCTTATACTGTGAAACTGCGAATGGCTCATTAAATCAGTTATAGTTTATTTGATG
GTACCTTTACTCGGATAACCGTAGTAATTCTAGAGCTAATACGTGCGTAAATCCCGACTTCTGGAAGGG
ACGTATTTATTAGATAAAAGGCCAGCCGGGCTTGCCCGACTCTTGGCGAATCATGATAACTTCACGAAT
CGCACGGCTTCGTGCCGGCGATGTTTCATTCAAATTTCTGCCCTATCAACTTTCGATGGTAGGATAGAG
GCCTACCATGGTGGTAACGGGTGACGGAGGATTAGGGTTCGATTCCGGAGAGGGAGCCTGAGAAACG
GCTACCACATCCAAGGAAGGCAGCAGGCGCGCAAATTACCCAATCCCAACACGGGGAGGTAGTGACA
ATAAATAACAATACCGGGCATTTTTGTCTGGTAATTGGAATGAGTACAATCTAAATCCCTTAACGAGTA
TCCATTGGAGGGCAAGTCTGGTGCCAGCAGCCGCGGTAATTCCAGCTCCAATAGCGTATATTTAAGTTG
TTGCAGTTAAAAAGCTCGTAGTTGGATTTCGGATGGGTTGTAGCGGTCAGCCTTTGGTGAGTACTGCTA
CGGCCCATCTTTCTGCCGGGGACGTGCTCCTGGGCTTAACTGTCCGGGACACGGAATCGGCGAGGTTAC
TTTGAGTAAATTAGAGTGTTCAAAGCAAGCCTACGCTCTGAATACATTAGCATGGAATAACACGATAG
GACTCTGGCTTATCTTGTTGGTCTGTAAGACCGGAGTAATGATTAAGAGGGACAGTCGGGGGCATTCGT
ATTTCATTGTCAGAGGTGAAATTCTTGGATTTATGAAAGACGAACTTCTGCGAAAGCATTTGCCAAGGA
TGTTTTCATTAATCAAGAACGAAAGTTGGGGGCTCGAAGACGATTAGATACCGTCGTAGTCTCAACCA
TAAACGATGCCGACTAGGGATTGGCAGGTGTTTCGTTGATGACCTGCCAGCACCTTATGAGAAATCAA
GTTTTGGGTTCGGGGGGAGTATGGTCGCAGGCTGAAACTTAAGGGAATTGACGGAAGGGCCCACCAGG
CGGGGAACCGGCGGTTATTTGACTCCACCGGGAAAATTTCCGGCCCAAACCGGGGGGAAATAAAAAAT
TAAACTTTTTGTTTTTTGGGGGGGGGGGGGGGGCCCCTTTTTTTGGGGGGGGGCCCGCGG

> #101_18S
GGAAGCTGCTCAAGATTAAGCCATGCATGTCTAAGTATAAACTGCTTATACTGTGAAACTGCGAATGG
CTCATTAAATCAGTTATAGTTTATTTGATGGTACCTTTACTCGGATAACCGTAGTAATTCTAGAGCTAAT
ACGTGCGTAAATCCCGACTTCTGGAAGGGACGTATTTATTAGATAAAAGGCCAGCCGGGCTTGCCCGA
CTCTTGGCGAATCATGATAACTTCACGAATCGCACGGCTTCGTGCCGGCGATGTTTCATTCAAATTTCT
GCCCTATCAACTTTCGATGGTAGGATAGAGGCCTACCATGGTGGTAACGGGTGACGGAGGATTAGGGT
TCGATTCCGGAGAGGGAGCCTGAGAAACGGCTACCACATCCAAGGAAGGCAGCAGGCGCGCAAATTA
CCCAATCCCAACACGGGGAGGTAGTGACAATAAATAACAATACCGGGCATTTTTGTCTGGTAATTGGA
ATGAGTACAATCTAAATCCCTTAACGAGTATCCATTGGAGGGCAAGTCTGGTGCCAGCAGCCGCGGTA
ATTCCAGCTCCAATAGCGTATATTTAAGTTGTTGCAGTTAAAAAGCTCGTAGTTGGATTTCGGGTGGGT
TGTAGCGGTCAGCCTTTGGTGAGTACTGCTACGGCCCACCTTTCTGCCGGGGACGTGCTCCTGGGCTTA
ACTGTCCGGGACACGGAATCGGCGAGGTTACTTTGAGTAAATTAGAGTGTTCAAAGCAAGCCTACGCT
CTGAATACATTAGCATGGAATAACACGATAGGACTCTGGCTTATCTTGTTGGTCTGTAAGACCGGAGTA
ATGATTAAGAGGGACAGTCGGGGGCATTCGTATTTCATTGTCAGAGGTGAAATTCTTGGATTTATGAAA
GACGAACTTCTGCGAAAGCATTTGCCAAGGATGTTTTCATTAATCAAGAACGAAAGTTGGGGGCTCGA
AGACGATTAGATACCGTCGTAGTCTCAACCATAAACGATGCCGACTAGGGATTGGCAGGTGTTTCGTT
GATGACCCTGCCAGCACCTTATGAGAAATCAAGCTTTTGGGTTCCGGGGGAAGTATGGTCGCAAGGCT
GAAACTTAAAGGAATTGACGGAAGGCCCCACCAGGGGTGGAGCCTGCGGCTTATTTGACTCACACGGG
AAAACTTACCAGGTCCAAACGGGGAAGAATGACAAATTGAAGCTCTTCCTGATCTGGGGGGAGGGGC
ATGCCGTCTTACCGTGGGGTGCCTGTCCAGGTGATTCCGGAACCAGCAACCCCAACCAAAAGAAACCC
CTTCTCCCGGGGGCCGGGTCTTAAACCAATGGGGGTACCCATGAGGGGGGGGAAAACCCTCGGGAATC
TTTTTTCCTGTCCCGCCCCCCCATTTTCTTGGGAACTCCCCGCGAGAGACGGGCTTTTTTATGTCTTGGG
GCATATTCTTAT

> #127_18S
GGAAAGCTGTCTCAAGATTAAGCCATGCATGTCTAAGTATAAACTGCTTATACTGTGAAACTGCGAAT
GGCTCATTAAATCAGTTATAGTTTATTTGATGGTACCTTTACTCGGATAACCGTAGTAATTCTAGAGCT

AATACGTGCGTAAATCCCGACTTCTGGAAGGGACGTATTTATTAGATAAAAGGCCAGCCGGGCTTGCC
CGACTCTTGGCGAATCATGATAACTTCACGAATCGCACGGCTTCGTGCCGGCGATGTTTCATTCAAATT
TCTGCCCTATCAACTTTCGATGGTAGGATAGAGGCCTACCATGGTGGTAACGGGTGACGGAGGATTAG
GGTTCGATTCCGGAGAGGGAGCCTGAGAAACGGCTACCACATCCAAGGAAGGCAGCAGGCGCGCAAA
TTACCCAATCCCAACACGGGGAGGTAGTGACAATAAATAACAATACCGGGCATTTTTGTCTGGTAATT
GGAATGAGTACAATCTAAATCCCTTAACGAGTATCCATTGGAGGGCAAGTCTGGTGCCAGCAGCCGCG
GTAATTCCAGCTCCAATAGCGTATATTTAAGTTGTTGCAGTTAAAAAGCTCGTAGTTGGATTTCGGGTG
GGTTGTAGCGGTCAGCCTTTGGTGAGTACTGCTACGGCCCACCTTTCTGCCGGGGACGTGCTCCTGGGC
TTAACTGTCCGGGACACGGAATCGGCGAGGTTACTTTGAGTAAATTAGAGTGTTCAAAGCAAGCCTAC
GCTCTGAATACATTAGCATGGAATAACACGATAGGACTCTGGCTTATCTTGTTGGTCTGTAAGACCGGA
GTAATGATTAAGAGGGACAGTCGGGGGCATTCGTATTTCATTGTCAGAGGTGAAATTCTTGGATTTATG
AAAGACGAACTTCTGCGAAAGCATTTGCCAAGGATGTTTTCATTAATCAAGAACGAAAGTTGGGGGCT
CGAAGACGATTAGATACCGTCGTAGTCTCAACCATAAACGATGCCGACTAGGGATTGGCAGGTGTTTC
GTTGATGACCCTGCCAGCACCTTATGAGAAACCAAAGTTTTTGGCTTCCGGGGGGAGTATGGTCGCAG
GCTGAAACTTAAAGGGATGGGACGGAAGGGCACACCAGGCGGAGCCTGCGCTTATTGCTTCAACCGG
GAAAACTATCAGCCCAGAACCGGGGAGCATGACGAATGAAGCTCTTTCTGGACCCGAGACGGCGCGCC
AGACCACCAAACCGCTGCGTTAACGTTCCGGATCATGTTGCTACCCCCCTACTTCAATCTTTAGGAAAC
CCCCTCCCCGTGCGGGGAGATCCTAT

> *D.tertiolecta*_18S
GAGCCATGCATGTCTAGTATAAACTGCTTATACTGTGAAACTGCGAATGGCTCATAAATCAGTTATAGT
TTATTTGATGGTACCTTTACTCGGATAACCGTAGTAATTCTAGAGCTAATACGTGCGTAAATCCCGACT
TCTGGAAGGGACGTATTTATTAGATAAAAGGCCAGCCGGGCTTGCCCGACTCTTGGCGAATCATGATA
ACTTCACGAATCGCACGGCTTTATGCCGGCGATGTTTCATTCAAATTTCTGCCCTATCAACTTTCGATGG
TAGGATAGAGGCCTACCATGGTGGTAACGGGTGACGGAGGATTAGGGTTCGATTCCGGAGAGGGAGC
CTGAGAAACGGCTACCACATCCAAGGAAGGCAGCAGGCGCGCAAATTACCCAATCCCAACACGGGGA
GGTAGTGACAATAAATAACAATACCGGGCATTTTTGTCTGGTAATTGGAATGAGTACAATCTAAATCCC
TTAACGAGTATCCATTGGAGGGCAAGTCTGGTGCCAGCAGCCGCGGTAATTCCAGCTCCAATAGCGTA
TATTTAAGTTGTTGCAGTTAAAAAGCTCGTAGTTGGATTTCGGGTGGGTTGTAGCGGTCAGCCTTTGGT
TAGTACTGCTACGGCCTACCTTTCTGCCGGGGACGAGCTCCTGGGCTTAACTGTCCGGGACTCGGAATC
GGCGAGGTTACTTTGAGTAAATTAGAGTGTTCAAAGCAAGCCTACGCTCTGAATACATTAGCATGGAA
TAACACGATAGGACTCTGGCTTATCTTGTTGGTCTGTAAGACCGGAGTAATGATTAAGAGGGACAGTC
GGGGGCATTCGTATTTCATGTCAGAGGTGAAATTCTTGGATTTATGAAAGACGAACTTCTGCGAAAGC
ATTTGCCAAGGATGTTTTCATTAATCAAGAAC

Table S3: FASTA sequence of the ITS gene sequence of tested *Dunaliella* strains

>#006_ITS

AGCCCCGTAACTTTTGGATTCTTATACCGCTGCCCTTCAGAAACAACTGAGCGATTGCTGCCTAACCGT
TTGGGTCCCTTGACGGGTAGCCGAGATAGCCCCTGCTCTTCAGCTGATCCTGATTCTTTGTTGGGGCCT
GAATCACCCAAGCTCTGGAACAGCCAGGTCCACTTACGTTATTCCTCACATGAGGGAGTGGTTCCTCCT
GTTAAAGAAGAAGGTTAAGGGTGGTTCCTTTGTTCAGCCAGACTGGATCTTCACCCAATAGTGAGGGA
GGAGGCTATCATACTTTCTGGTTCGCATCTTTGTTTGTTGCGAAGTGCTACCCCGGTGAAGATTT

>#27_ITS
TGATGATTCACGGAATTCTGCAATTCACACTACGTATCGCATTTCGC

>#37_ITS
TCTTAGGTTTTGGAGGGCCAAGCCCATGGTCCCAAGCCAACAACTAGAAATAGCGAGCGATTGCTGCC
TACCCAGTTGCGGCCCTTGACGGGTCCTTTGAGCTAGCCTCTGCTCTTCAGCTGATCCAGAGCCTTTGTT
GGGGCAGTGAAGCACCCAAGCTCTGGAACAGCCAGGTCCACTAACATTACTCCTCACATGGGGGAGTG
GTTAGTGAGATTAACCCGACGCTGAGGCAAACATGCCCTTGGCCGAAGCCGCGGACGCAATTTGCGTT
CAAAGATTTGATGATTCACGGAATTCTGCAATTCACACTACGTATCGCATTTCGCTGCGCCTCTTCACC
GAAGCGCCA

>#83_ITS
GCTTCGGTCGAGAAAAGAAGGGTTCCTGTTTGAGGGCCAAGCCCATGGTCCCAAGCCAACAACTAGAA
ATAGCGAGCGATTGCTGCCTACCCAGTTGCGGCCCTTGACGGGTCCTTGAGCTAGCCTCTGCTCTTCAG
CTGATCCAGAGCCTTTGTTGGGGCAGTGAAGCACCCAAGCTCTGGAACAGCCAGGTCCACTAACATTA
CTCCTCACATGGGGGAGTGGTTAGTGAGATTAACCCGACGCTGAGGCAAACATGCCCTTGGCCGAAGC
CGCGGACGCAATTTGCGTTCAAAGATTTGATGATTCACGGAATTCTGCAATTCACACTACGTATCGCAT
TTCGCTGCGTTCTTAATCA

>#96_ITS
GAGCTCAGGTCGAGAAAAAAAGAGGTTCCTGTTTGAGGGCCGAGCCCATGGTCCCAAGCCAACAACTA
GAAATAGCAAGCGTGAGCTGCCTACCCAGTTGCGGCCCTTGACGGGTCCTTGAGCTAGCCTCTACTCTT
CAGCTGATCCAGAGCTTTTGCTAAGCGCAATGAAGCACTCAAGCTCTGGAACAGCCAGGTCCACTTAC
CCGCTTCCACTATGGGAGGAGGGGAGTGAGATTAACCCGACGCTGAGGCAAACATGCCCTCAGCCGAA
GCCTTGGGCGCAATTTGCGTTCAAAGATTTGATGATTCACGGAATTCTGCAATTCACACTACGTATCGC
ATTTCGCTGCGTTTTA

>#*D.tertiolecta*_ITS
TCTGCTTGAGCTCAGGTCGAGAAAAAAAGAGGTTCCTGTTTGAGGGCCGAGCTCATGGTCCCAAGCCA
ACAACTAGAAATAGCAAGCGTGAGCTGCCTACCCAGTTGCGGCCCTTGACGGGTCCTTGAGCTAGCCT
CTACTCTTCAGCTGATCCAGAGCTATTGCCAAGCGCAATGAAGCACTCAAGCTCTGGAACAGCCAGGT
CCACTTACCCGCTTCCACTATGGGAGGAGGGGAGTGAGATTAACCCGACGCTGAGGCAAACATGCCCT
CAGCCGAAGCCTTGGGCGCAATTTGCGTTCAAAGATTTGATGATTCACGGAATTCTGCAATTCACACTA
CGTATCGCATTTCGCTGCGTTCTTAAT

Table S4: NCBI blast results for the 18S sequence of the *Dunaliella* isolates.

Isolate	Strain	Accession	Identity (%)
Dunaliella sp. 6	*Dunaliella salina*	AF506698.1	95.62
	Dunaliella sp. SPMO 201-8	DQ324014.1	95.33
	Dunaliella salina strain KU13	KF825552.1	95.26
	Dunaliella salina strain UTEX LB 1644	DQ009765.1	95.12
Dunaliella sp. 27	*Dunaliella polymorpha* strain ST10.1	MN167111.1	99.10
	Dunaliella polymorpha	KY923056.1	99.10
	Dunaliella sp. SPMO 601-1	DQ324021.1	99.10
	Dunaliella viridis strain CONC002	DQ009776.1	99.10
Dunaliella sp. 37	*Dunaliella salina* strain UU1	KX377708.1	95.65
	Dunaliella sp. SPMO 601-1	DQ324021.1	95.59
	Dunaliella viridis strain CONC002	DQ009776.1	95.59
	Dunaliella sp. MBTD-CMFRI- S147	JN807320.2	95.58
Dunaliella sp. 83	*Dunaliella polymorpha* strain ST10.1	MN167111.1	98.15
	Dunaliella polymorpha	KY923056.1	98.15
	Dunaliella parva	KT355034.1	98.15
	Dunaliella polymorpha strain CCAP 19/14	KJ756825.1	98.15
Dunaliella sp. 96	*Dunaliella polymorpha* strain ST10.1	MN167111.1	99.18
	Dunaliella polymorpha	KY923056.1	99.18
	Dunaliella parva 18S	KT355034.1	99.18
	Dunaliella polymorpha strain CCAP 19/14	KJ756825.1	99.18
Dunaliella sp. 101	*Dunaliella salina* strain UU1	KX377708.1	97.50
	Dunaliella viridis strain UTEX 1644	KJ018726.1	97.46
	Dunaliella sp. MBTD-CMFRI-S147	JN807320.2	97.40
	Dunaliella primolecta strain CCAP 11/34	KJ756819.1	97.01
Dunaliella sp. 127	*Dunaliella parva*	KT355034.1	99.29
	Dunaliella polymorpha strain CCAP 19/14	KJ756825.1	99.29
	Dunaliella parva	M62998.1	99.29
	Dunaliella salina strain UU1	KX377708.1	97.93

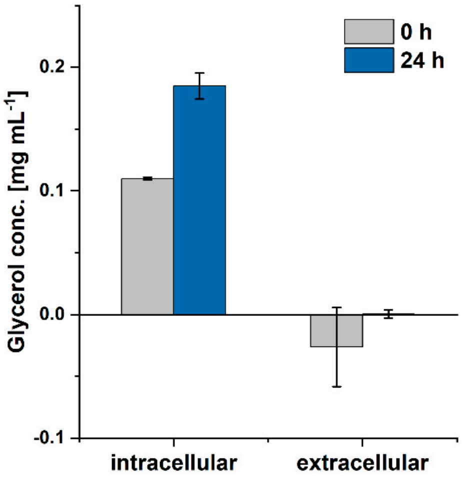

Figure S1: Glycerol concentration of intracellularly and extracellularly derived glycerol, extracted from *D. tertiolecta* after cultivation at 28 °C in modified Johnson medium (pH = 7.5) containing 1 M NaCl. After 14 days, the NaCl concentration was increased to 2 M, and glycerol concentration was measured 0 h and 24 h after hyperosmotic change.

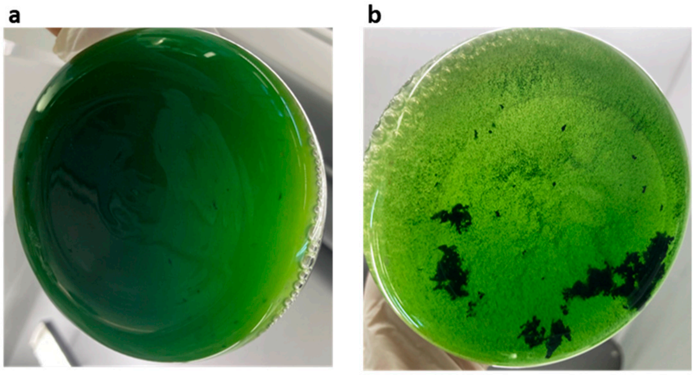

Figure S2 a) Bottom view of the flask of *Dunaliella* sp. 96 after one week of cultivation in 1 M NaCl containing modified Johnson medium (pH=7.5) at 28°C. b) Bottom view of the flask of *Dunaliella* sp. 96 after one week of cultivation in 1.5 M NaCl containing modified Johnson medium, demonstrating the forming of aggregated cells.

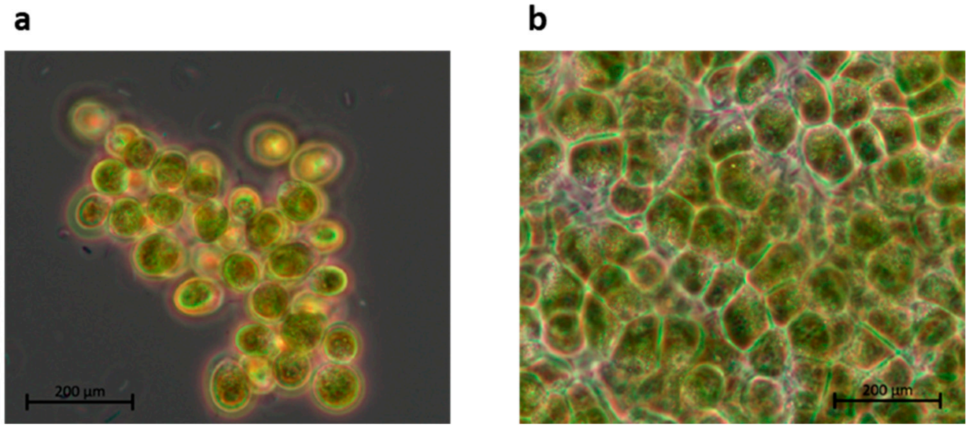

Figure S3: Bright field images (20x) of *Dunaliella* sp. 96 when cultivated in a) 1.5 M and b) 2 M NaCl containing modified Johnson medium (pH=7.5). The higher the salt concentration, the earlier the cells start to aggregate and the larger the formed palmella structures.
